# Granzyme B + CD8 + T cells with terminal differentiated effector signature determine multiple sclerosis progression

**DOI:** 10.1186/s12974-023-02810-0

**Published:** 2023-06-02

**Authors:** Ziyan Shi, Xiaofei Wang, Jiancheng Wang, Hongxi Chen, Qin Du, Yanlin Lang, Lingyao Kong, Wenqin Luo, Yuhan Qiu, Ying Zhang, Chen Li, Dingke Wen, Jie Yao, Xia Cheng, Linjun Cai, Xue Lin, Rui Wang, Zichao Mou, Shuangjie Li, Duanya Liu, Hong Zhou, Hongyu Zhou, Mu Yang

**Affiliations:** 1 Department of Neurology, West China Hospital, Sichuan University, No.28 Dianxin Nan Street, Chengdu, 610041 China; 2 Centre for Translational Research in Cancer, Sichuan Cancer Hospital and Institute, No.55 South Renmin Road, Chengdu, 610000 China; 3 School of Medicine, University of Electronic Science and Technology of China, Chengdu, 610000 China; 4 Department of Neurosurgery, West China Hospital, Sichuan University, Chengdu, 610041 China; 5 School of Life Science and Technology, University of Electronic Science and Technology of China, Chengdu, 610000 China

**Keywords:** Multiple sclerosis, Secondary progression, Single-cell RNA sequencing, CD8 + T_EMRA_ cells, GzmB, Clonal expansion

## Abstract

**Background:**

Multiple sclerosis (MS) leads to demyelination and neurodegeneration with autoimmune responses in central nervous system. Patients begin with a relapsing–remitting (RR) course, and more than 80% of them may advance to secondary progressive MS (SPMS), which is characteristic for the gradual decline of neurological functions without demonstrated treating method to prevent. This study aims to investigate the contribution of peripheral CD8 + T cells during the conversion from RRMS to SPMS, as well as reveal potential diagnostic signature in distinguishing SPMS.

**Methods:**

Single-cell RNA sequencing was employed to reveal the heterogeneity of CD8 + T cells between SPMS and RRMS. In addition, flow cytometry was used to further characterized CD8 + T cell dynamic changes in patients. T cell receptor sequencing was performed to detect the clonal expansion of MS. Using *Tbx21* siRNA, T-bet was confirmed to manipulate GzmB expression. The correlation between GzmB + CD8 + T cell subsets and clinical characteristics of MS and their potential diagnostic value for SPMS were evaluated by generalized linear regression models and receiver operating characteristic (ROC) curve respectively.

**Results:**

Other than diminished naïve CD8 + T cell, elevating of activated CD8 + T cell subsets were observed in SPMS patients. Meanwhile, this aberrant amplified peripheral CD8 + T cells not only exhibited terminal differentiated effector (EMRA) phenotype with GzmB expression, but also possessed distinct trajectory from clonal expansion. In addition, T-bet acted as a key transcriptional factor that elicited GzmB expression in CD8 + T_EMRA_ cells of patients with SPMS. Finally, the expression of GzmB in CD8 + T cells was positively correlated with disability and progression of MS, and could effectively distinguish SPMS from RRMS with a high accuracy.

**Conclusions:**

Our study mapped peripheral immune cells of RRMS and SPMS patients and provided an evidence for the involvement of GzmB + CD8 + T_EMRA_ cells in the progression of MS, which could be used as a diagnostic biomarker for distinguishing SPMS from RRMS.

**Supplementary Information:**

The online version contains supplementary material available at 10.1186/s12974-023-02810-0.

## Background

As a chronic inflammatory disease of central nervous system (CNS), multiple sclerosis (MS) leads to demyelination and neurodegeneration with autoimmune responses [1]. In most of the cases, MS begins with a relapsing–remitting (RR) course, and more than 80% of patients with RRMS may advance to secondary progressive MS (SPMS), which is characteristic for the gradual decline of neurological functions without relapse [2]. Although currently disease-modifying therapies (DMT) have lengthened the period from relapse onset to secondary progression phase, but no treatment with demonstrated efficacy are found to prevent the worsen of SPMS [3, 4]. Meanwhile, less diagnostic markers or therapeutic targets for disease progression are determined on account of the undefined mechanism driving transition from RRMS to SPMS [5, 6].

In clinical practice, continuous early increasing of Expanded Disability Status Scale (EDSS) scores, or scores changed from baseline up to 24 months are considered as hallmarks of SPMS [7]. Results from routine neurological inspections, such as gadolinium enhancement and higher T2 lesion burden from MRI could only be evidences for symptomatic MS [8, 9]. Therefore, owing to obtain obvious image evidences and/or long-term clinical evaluation, progressed patients still need to accumulate minimum levels of disability for a diagnosis to be made [7, 10]. Indeed, most of SPMS patients exhibit atypical symptoms at the initial stage, once prominent symptoms manifest, the CNS has already been taxed in many cases [6]. Pathophysiologically, it remains debatable whether less blood–brain barrier (BBB) permeability, inflammation versus neurodegeneration, as well as macrophages/microglia polarization could be the features to differ SPMS and RRMS [11–13]. According to the merging view that RRMS and SPMS are part of a disease continuum with an indistinct boundary, no prominent factor for predicting progression to SPMS in patients with RRMS [14]. Another limitation of histopathological studies is the direction of causality requires prospective assessment, which is not feasible for human nervous tissues [10]. Up to date, due to inexorable and incurable progression of disabilities in SPMS patients, there is a critical clinical need for identification of the conversion from RRMS to SPMS at early stage.

Addition to CD4 + T cells trigger experimental autoimmune encephalomyelitis (EAE) in murine model, as well as clonal expansion of B lymphocytes and plasma cells in MS patients at active stages, provoked CD8 + T cells are observed as dominant population over all lymphocyte subsets at the lesion sites in progressive MS [15, 16]. Due to prominent association between viral infection and MS onset, latest studies report that activated memory CD8 + T cells may be responsible for demyelination and axonal damage in SPMS [17, 18]. Besides, we previously revealed that effector/memory (EM) CD8 + T cell proportion significantly elevates in peripheral of patients with MS [19]. These CD8 + T_EM_ cells synergizing with macrophages are capable of mediating autoimmune peripheral neuropathy, which shares similar pathogenesis of Gillian-Barre Syndrome [20]. Here, to further investigate whether immune cascades mediated by peripheral CD8 + T cells in contributing SPMS transition, single-cell RNA sequencing (scRNAseq) was employed to differ the heterogeneity between peripheral CD8 + T cells from patients with RRMS or SPMS. Trajectories of CD8 + T cell expansion were drawn for uncovering alternative effector differentiation in comply with clonal expansion to contribute MS progression as well. In addition, Granzyme B (GzmB) + terminal differentiated effector (EMRA) CD8 + T cells were determined to elicit autoreactive immune responses thus give rise the transition of MS in patients.

## Materials and methods

### Participants

All MS patients in this study were from West China Hospital of Sichuan University and met the 2017 revisions of McDonald criteria [21]. Age- and sex-matched healthy donors (HD) were also enrolled. Peripheral blood samples from all participants were collected between January 2021 and May 2022. The diagnosis of SPMS was based on the neurological deterioration in the absence of relapse lasting more than 6 months after the relapsing–remitting course [7]. EDSS scores were assessed for each patient with MS to evaluate the disability. “Progressive” state was defined as EDSS scores increase 1-point with an EDSS score ≤ 5.5 or increase 0.5 point with an EDSS score ≥ 6.0 during the past year and the “stable” state was defined as without any change of EDSS score [7, 22]. Demographic and clinical characteristics were summarized in Table 1. This study was approved by the Medical Ethics Committee of the West China Hospital, Sichuan University and all participants given informed consent prior to their inclusion in this study.

**Table 1:** Demographic and clinical characteristics of participants

	HD n = 24	RRMS n = 30	SPMS n = 20	*P*-value
Female, n (%)	16 (67%)	23 (77%)	11 (55%)	0.275
Age, median (IQR), years	33 (26-53)	32 (27-38)	36 (32-46)	0.057
Age at onset, median (IQR), years		27 (24-32)	29 (21-38)	0.841
Disease duration, median (range), years		2.2 (1.2-5.5)	9.8 (3.7-11.5)	< 0.001
Status, n (%)				
Acute attack (< 1 months)		5 (16%)	1 (5%)	0.139
Non-acute attack (≥ 1 months)		25 (84%)	19 (95%)	
Treatments, n (%)				
Untreated		16 (53%)	11 (55%)	0.908
Treated^a^		14 (47%)	9 (45%)	
OCB, positive, n(%)		19/25 (76%)	16/20 (80%)	0.519
EDSS score, median(IQR)		1.5 (1.0-2.1)	6.0 (4.0-6.0)	< 0.001

HD = Healthy donors; RRMS = Relapse-remission multiple sclerosis; SPMS = Secondary progressive multiple sclerosis; IQR = Interquartile range; OCB = Oligoclonal immunoglobulin G bands, EDSS = Expanded Disability Status Scale; T25W = Timed-25-foot walk test; MSWS-12 = 12-Item Multiple Sclerosis Walking Scale; 9-HT= 9-Hole Peg Test

^a^Treatments include β-IFN, Teriflunomide, and/or Corticosteroids

### Processing of single-cell RNA sequencing (scRNA-seq) data

Peripheral blood mononuclear cells (PBMCs) were isolated using Ficoll–Paque PLUS (GE) according to the manufacture’s protocol. Red blood cells were lysed by ACK buffer (Gibco) and filtered through a 40 μm filter after Ficoll isolation. Single cell suspensions were loaded into 10 × Genomic to capture approximately 8000 cells according to the manufacturer’s instructions of 10X Genomics Chromium Single-Cell V(D)J kit (V5). Single-cell libraries were sequenced on an Illumina NovaSeq 6000 sequencing system (paired-end multiplexing run, 150 bp) by LC-Bio Technology co.ltd., (China) at a minimum depth of 20,000 reads per cell. Samples were integrated and analyzed using *Seurat* package (v4.0.6) in R software (v4.0.2) [23]. The batch effect was adjusted using *harmony* (v0.1.0) [24]. Identities of clusters were manually annotated using well-recognized cell markers according to published articles [25]. Gene set variation analysis (*GSVA* package, v1.32.0) was performed to compare the functional profiles of different cell clusters, and the annotation gene sets were downloaded from C5 category (GO:BP) of the molecular signature database (MSigDB, version 7.0) [26, 27]. Pseudotime trajectory analysis was performed using *Monocle2* package (v2.14.0) based on DDRTree method [28].

### Processing of T cell receptor (TCR) data

The single-cell V(D)J sequences were processed using cellranger and annotated based on GRCh38 reference from Ensembl database. Using *LymphoSeq* (v1.16.0) package [29], unproductive TCR rearrangements were filtered out. The clonal diversity was calculated using Shannon Entropy and Gini Coefficient, both indexes were reported on a scale from 0 to 1, where 0 indicates all TCR sequences have the same frequency, and 1 indicates the TCR repertoire is dominated by a single sequence. Then, the prevalence of productive TCR sequences in 55 PBMCs of healthy donors was calculated (github.com/davidcoffey/LymphoSeqDB). MS-specific TCR repertoire was sorted if the prevalence of the TCR sequence was 0 in healthy donors. TCR clonotypes with absolute counts > 500 were defined as expanded clonotypes based on the overall distribution of MS-specific TCR repertoires. The expanded clonotypes were then projected to scRNA-seq data based on the same cell barcode using *‘AddMetaData’* function of *Seurat* package. The expanded TCR clonotypes of MS were annotated in VDJdb, a curated database of T-cell receptor (TCR) sequences with known antigen specificities. The potential epitopes for a TCR to recognize was predicted based on amino acid sequences of complementarity determining region-3 (CDR3).

### Flow cytometry assay

Peripheral blood samples from MS patients were prepared to stain for flow cytometry assay as previously studies [19]. PBMCs were stained with selected antibodies for 30 min after incubated with Human TruStain FcX™ (Biolegend) at 4°. Anti-human CD3-APC, CD8a-PerCP, CD45RA-PE, and CCR7-APC/Cyanine7 were used to label surface markers of CD8 + T cells. Granzyme B-FITC, T-bet-PE/Cyanine7 and Eomes-PE were selected for Intracellular staining after fixation and permeabilization by Foxp3/Transcription factor staining buffer (Invitrogen). FACS Canto II flow cytometer and Flowjo v10 (BD) were employed to obtain the original data and perform further analysis, respectively.

### Tbx21 knockdown

Accell Human *Tbx21* siRNA SMARTpool and Non-Targeting Control (NC) Pool were purchased from Dharmacon. Magnetic isolation was performed to isolate CD8 + T cells from PBMCs of SPMS patients using MojoSort™ Human CD8 T Cell Isolation Kit (Biolegend). CD8 + T cells were co-cultured with 1 μmol/L *Tbx21* siRNA or NC siRNA for 96 h, respectively, and flow cytometry assay was used to detect the protein levels of T-bet as mentioned above. Total RNA was extracted from cultivated CD8 + T cells and reverse transcribed into cDNA (PrimeScript™ RT Regent Kit, Takara) after 72 h transfection. Knockout efficiency of *Tbx21* was quantitatively assessed by qPCR (SYBR Premix Ex Taq II, Takara) in comparing with negative control (NC) and positive control (GAPDH) expression.

### Statistical analysis

Statistical calculations were performed using GraphPad Prism 8.0 (GraphPad Software) and/or SPSS software V25.0 (IBM Corp). Continuous variables were described by median and interquartile range (IQR). Categorical variables were shown as numbers and percentages. Mann–Whitney U test was used for continuous variable comparison between two groups, and Kruskal–Wallis with Dunn’s multiple comparisons were used for comparisons among three or more groups. Chi-square test was used to compare categorical variables between groups. The associations between GzmB levels in CD8 + T cell subsets and disability of MS were investigated with spearman correlation analysis. Generalized linear regression models were used to estimate the correlation of GzmB expression levels in CD8 + T cell subsets with clinical features (including sex, age, disease duration, EDSS scores, disease subtype, status, and treatments). A receiver operating characteristic (ROC) curve was established to evaluate the diagnostic accuracy of the GzmB in CD8 + T cell subsets for diagnosis of RRMS and SPMS. P values at two-tailed less than 0.05 were defined as statistically significant.

## Results

### Peripheral immune atlas in patients with MS

**Fig. 1:**
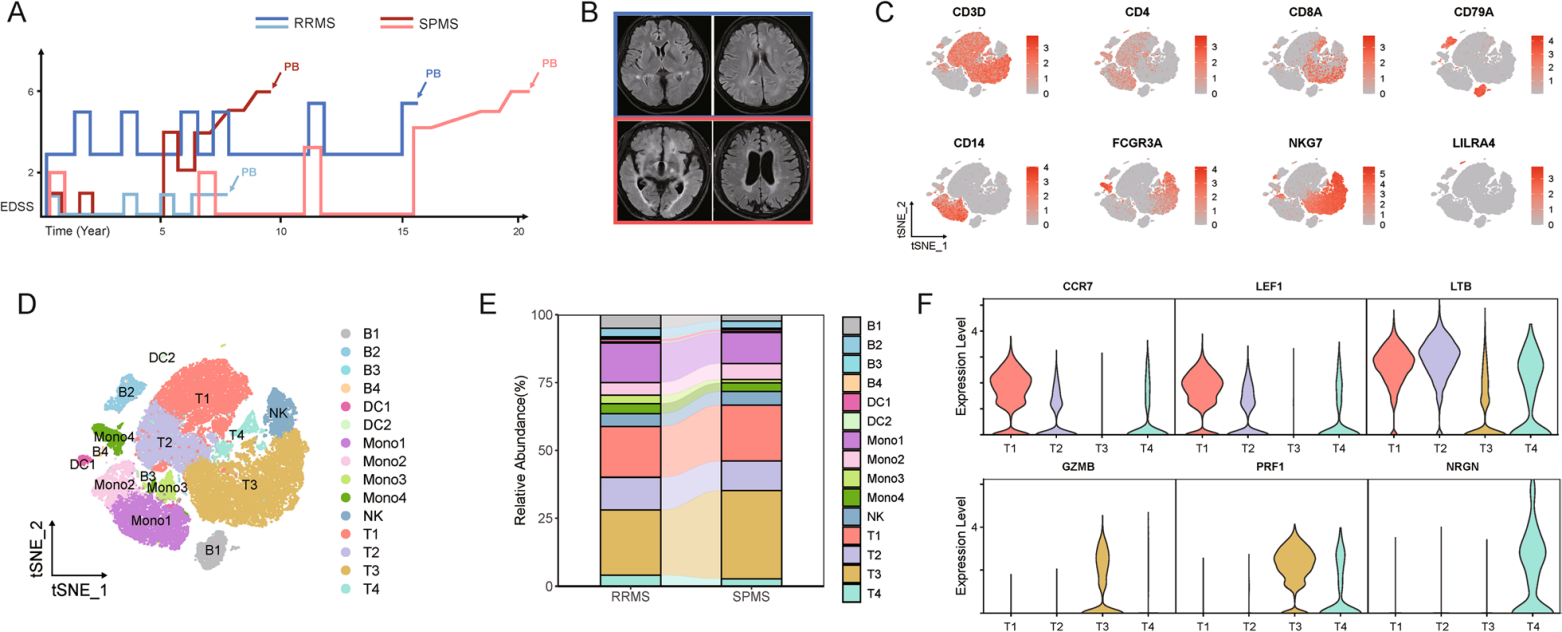
Single-cell RNA sequencing of peripheral blood in RRMS and SPMS patients. **A** Disease duration and EDSS scores of RRMS (*n* = 2) and SPMS (*n* = 2) patients used for single-cell RNA sequencing. **B** Representative MRI images (T2-weighted, FLAIR) of brain lesions of RRMS and SPMS patients. **C** Representative cell markers that used to define periphery immune cells. **D** T-SNE plot of periphery immune cells. **E** Comparison of the relative abundance of periphery immune cells between RRMS and SPMS. **F** Representative marker genes of T cell subclusters

Blood samples were collected from 2 RRMS patients with relapse stage and 2 SPMS patients at progressive stage, respectively, and all patients did not receive DMT or steroids in the past 6 months (Fig. 1A, B). After removal of red blood cells and low-quality cells, unsupervised method was applied to partition 35,834 single-cell transcriptomes into 15 clusters from scRNAseq analysis, including T/B cells, DCs, monocytes, and natural NK cells identified by unique gene signatures as follow: CD3D for T cells, CD79A for B cells, CD14 and FCGR3A for monocytes, NKG7 for NK cells, and LILRA4 for DCs (Fig. 1C and Additional file 1: Fig. S1). Of note, 4 distinct subclusters were observed in T cells, B cells and monocytes, respectively, whereas 2 segregated subclusters were found in dendritic cells, no subcluster was shown in NK subset (Fig. 1D). Besides, a small platelet cluster with promising PPBP expression was excluded for our further analysis (Fig. 1C and Additional file 1: Fig. S1). To distinguish dynamic changes of peripheral immune cells in mediating SPMS, relative abundance of each subclusters were generated between RRMS and SPMS (Fig. 1E). In comparing with RRMS patients, the most remarkably increasing was observed in T3 subcluster of SPMS patients (Fig. 1D, E). Further analysis confirmed that T3 subcluster substantially expressed GZMB and PRF1, which are characteristics of effector T cells (Fig. 1F). Other than that, the proportions of B1, B2 in B cell cluster and Mono1 subclusters from monocyte cluster were found to slightly decrease in patients with SPMS (Fig. 1E). Evidence from the Shared Nearest Neighbor algorithm analysis revealed that both B1 and B2 subclusters from SPMS patients were diminished with CD40, PAX5 and XBP, which are responsible for B cell maturation, as well as antigen-presenting abilities (Additional file 1: Fig. S2A, B). Taken together, elevating of peripheral effector T cell subclusters in SPMS patients may have an effect on discriminating MS with relapse and progressive states.

### Extensive heterogeneity of peripheral CD8 + T cells in patients with RRMS or SPMS

**Fig. 2:**
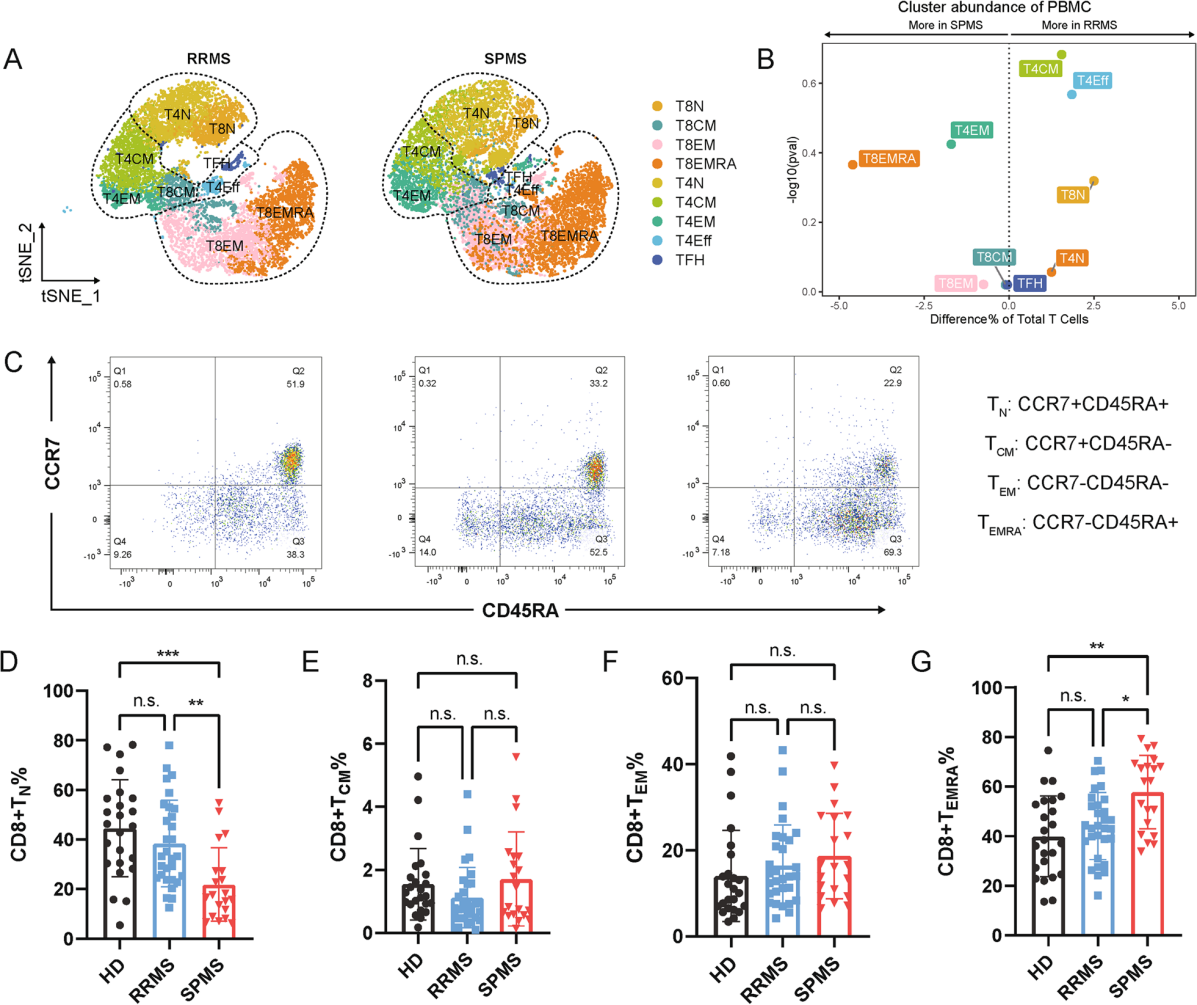
Increasing abundance of peripheral CD8 + T_EMRA_ in SPMS. **A** T-SNE plots of T cell subclusters in patients with RRMS and SPMS. **B** Comparison of the relative abundance of T cell subclusters between RRMS and SPMS. **C, D** Percentage of circulating CD8 + T cell subpopulations in HD, RRMS and SPMS. **C** The gating strategy of CD8 + T cell subpopulations for flow cytometry. **D–G** Percentages of T_N_, T_CM_, TEM, and T_EMRA_ of CD8 + T cells in HD (*n* = 24), RRMS (*n* = 30) and SPMS (*n* = 20). ***P < 0.001, **P < 0.01, *P < 0.05, n.s. (not significant)

To infer the potential phenotypes of effector T cells in triggering disease progression, we then subdivided T cell cluster into 9 subsets, including naïve (N), central memory (CM), EM, effector (Eff) and follicular helper (FH) CD4 + T cells, as well as N, CM, EM and EMRA CD8 + T cells (Fig. 2A and Additional file 1: Fig. S3). Notably, the most extensive heterogeneity of T cell subsets between RRMS and SPMS were CD8 + T_EMRA_ cells increasing and CD8 + T_N_ cell eliminating (Fig. 2B). Further identification was performed by collecting the peripheral blood samples from 50 MS patients (30 RRMS patients and 20 SPMS patients, respectively), as well as 24 healthy participants (healthy donor, HD) (Table 1), both CD8 + T_CM_ and T_EM_ cell proportions were consistent among 3 groups, whereas similar decreasing pattern of CD8 + T_N_ and increasing pattern of CD8 + T_EMRA_ cell patterns were observed in SPMS by comparing with HD and RRMS (Fig. 2C–G). Interestingly, the mildly elevating level of CD8 + T_EMRA_ cell proportion seemed not identical with the distantly segregated of T8EMRA cluster shown in abundance assay of scRNAseq, which may result from the discrepancy of individual patients (Fig. 2B, D, G). In addition, to understand the potential implication of treatments (β-IFN, Teriflunomide, and/or Corticosteroids) in CD8 + T cells phenotypes of MS, we compared untreated group with treated group. However, there is no significant changes of CD8 + T cell subsets was observed between untreated and treated groups in both RRMS and SPMS cohort (Additional file 1: Fig. S4). Overall, these findings indicated an aberrant amplified of CD8 + T_EMRA_ cells in the peripheral system of SPMS patients.

### Clonal expansion of peripheral CD8 + T cells in patients SPMS

**Fig. 3:**
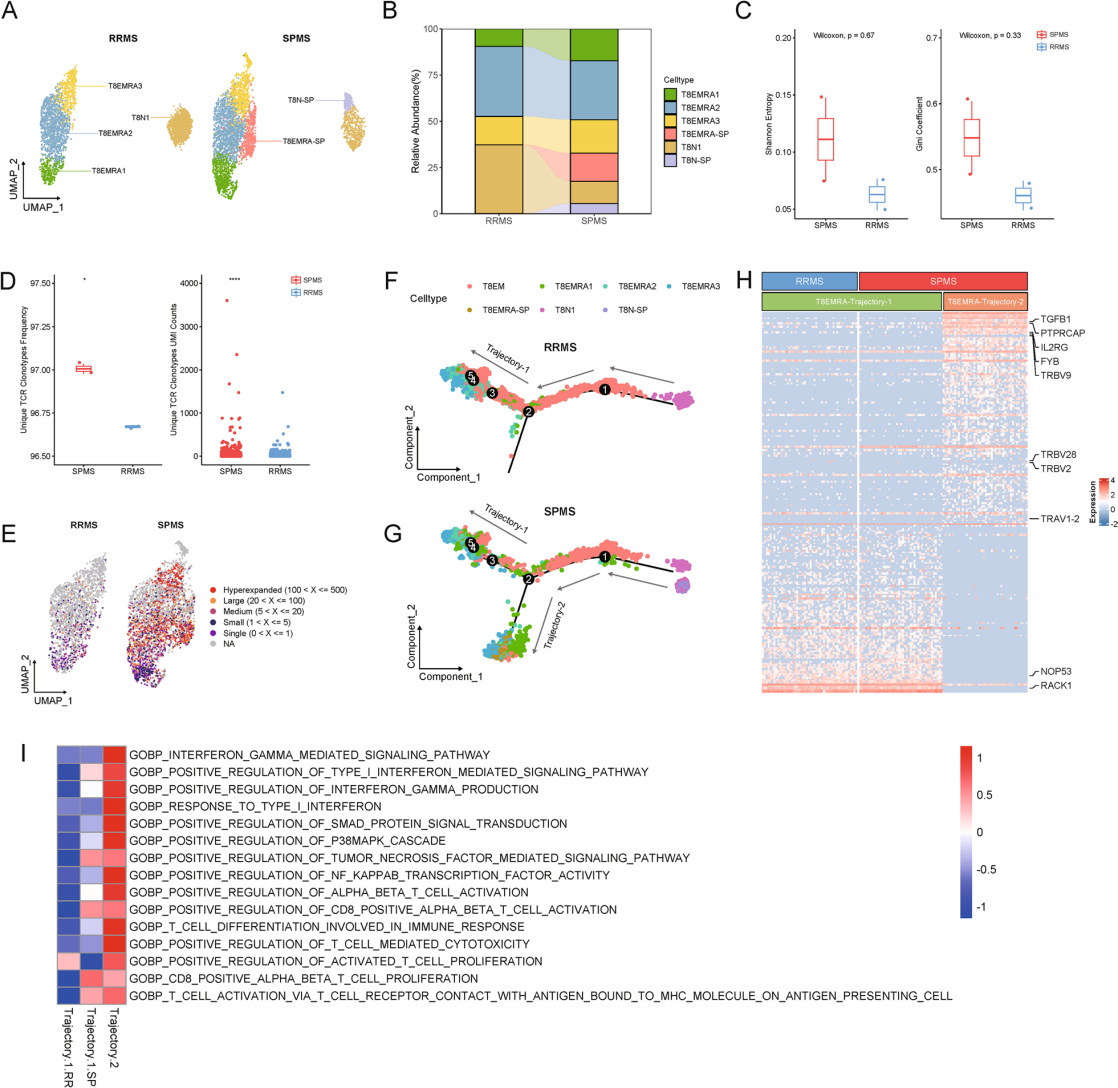
Clonal expansion and distinct trajectory of peripheral CD8 + T_EMRA_ in SPMS. **A** UMAP visualization of T_EMRA_ and T_N_ clusters. Patients with SPMS showed two unique clusters, T8EMRA-SP and T8N-SP. **B** SPMS showed obviously decreased proportion of Naïve CD8 + T cells and increased proportion of T_EMRA_ CD8 + T cells. **C** SPMS showed a decreasing trend in TCR diversity. **D** T cells from SPMS patients exhibited significantly clonal expansion. **E** The clonal expanded T cells exclusively mapped to CD8 + T_EMRA_ cells in SPMS. **F**, **G** Distinct trajectories of peripheral CD8 + T cells in RRMS and SPMS. **H** Heatmap plot of DEGs of T8EMRA-Trajectory-2 compared with T8EMRA-Trajectory-1. **I** Results of geneset variation analysis (GSVA) of GO: biological process database revealed the activating and/or effector states of T8EMRA-SP subclusters

To reveal the programming processes of peripheral CD8 + T cells, CD8 + T_N_ (T8N) and T_EMRA_ (T8EMRA) clusters were further divided into 6 populations (Fig. 3A). The abundances of T8N-SP, T8EMRA-SP, T8EMRA3 and T8EMRA1 subclusters were largely inflated, whereas T8N1 and T8EMRA2 subclusters were found to reduce in SPMS patients (Fig. 3B). Following, a comprehensive, genome-wide method was employed to uncover the specificity of CD8 T cell-derived TCRs from patients with MS (Fig. 3C–E). Comparison of patients with RRMS, CD8 T cells from SPMS patients were identified a decreasing trend in TCRs diversity (Fig. 3C). Notably, by excluding the overlapped TCRs in HD, those CD8 T cells exhibited significantly clonal expansion in SPMS, which might indicate an enhancement of antigen-specific immune responses (Fig. 3D). Moreover, the hyperexpanded CD8 T cells exclusively mapped to clusters of T8EMRA3 and T8EMRA-SP and were predominant in SPMS (Fig. 3E). Then, cell trajectories of each distinct cluster were revealed by constructing the pseudotime analysis and identified two distinct trajectories for TEMRA differentiation (Fig. 3F, G). In the first trajectory (Trajectory-1), shared by both RRMS and SPMS, cells originate from Naïve CD8 + T cells, gradually differentiate into T8EM cells, then sequentially become T8EMRA1/2/3 cells (Fig. 3F). In the second trajectory (Trajectory-2), which is only observed in SPMS, cells originate from two Naïve CD8 + T cell clusters and differentiate into T8EMRA1/2/3/SP cells. The cell fates of T8EMRA1/2/3 are different depending on which disease state they belong to, indicating diverse transcriptome profiles of the same cell clusters.

Meanwhile, other than elevated expression of TCR lineage-related genes that recognized TRBV9, TRBV28, TRVB2 and TRAV1-2, T8EMRA-SP subcluster with highly clonal expansion were also detected to bear increasing levels of markers associated with activated T cells, such as TGFB1, PTPRCAP, IL2RG, and FYB (Fig. 3H). It is worth to mention that apoptosis-related genes (NOP53, RACK1) were found to down-regulate in cells in Trajectory-2 as well (Fig. 3H). Following GSVA analysis indicated an enhancement of IFNγ and TNF-related pathways, suggesting the activating and/or effector states of T8EMRA-SP subclusters (Fig. 3I). In addition, annotation of clonal amplified TCR sequences showed that SPMS was associated with Cytomegalovirus (CMV) and Epstein-Barr virus (EBV) infection (Additional file 2: Table S1). Taken together, our results revealed a potentially dominant role of T8EMRA expansion in contributing antigen-specific inflammatory cascades, thus leading the pathogenesis of SPMS.

### High level of GzmB is associated with increasing numbers of CD8 + TEMRA cells in SPMS patients

**Fig. 4:**
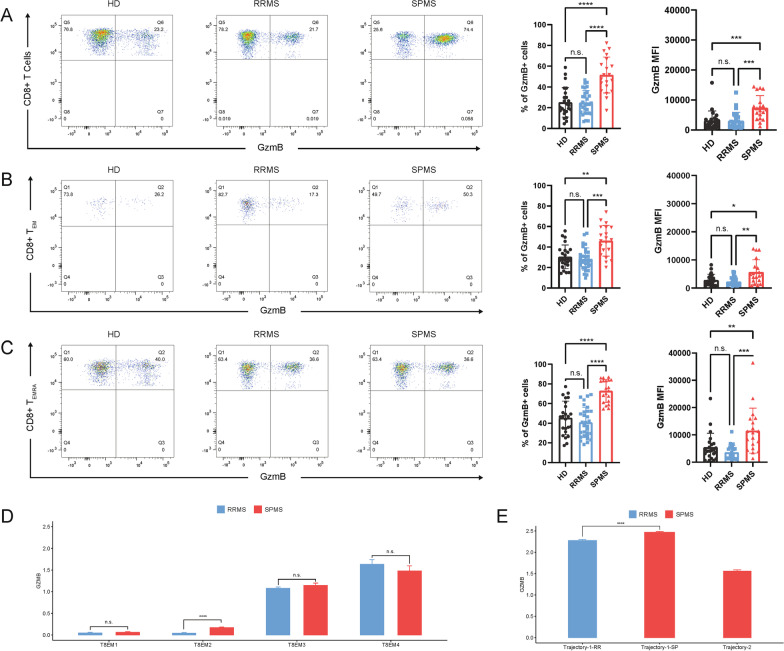
Increased expression of GzmB in CD8 + T cells in SPMS compared with RRMS. **A**–**C** The expression of GzmB in CD8 + T cell subpopulations was tested by flow cytometry. Peripheral blood from HD (*n* = 24), RRMS (*n* = 30) and SPMS (*n* = 20) were collected for testing. Frequencies and mean fluorescence intensity (MFI) of GzmB expression in CD8 + T cells (**A**), CD8 + T_EM_ (**B**), and CD8 + T_EMRA_ (**C**) were measured. **D**, **E** GzmB expression in T8EM and T8EMRA subclusters were analyzed using single-cell RNA sequencing data. ****P < 0.0001, ***P < 0.001, **P < 0.01, n.s. (not significant)

For determining the pathological function of CD8 + T cells in MS progression, GzmB level was measured in peripheral CD8 + T cells of our involved MS patients and HD, by comparing with HD and RRMS patients, GzmB level was found significant up-regulation in CD8 + T cells from SPMS patients (Fig. 4A). Following, the origin of GzmB enhancement was detected in CD8 + T_EM_ and CD8 + T_EMRA_ cells, respectively (Fig. 4B, C). Of note, in addition to both CD8 + T_EM_ and CD8 + T_EMRA_ cells exhibited excessive levels of GzmB, nearly all GzmB + CD8 + T cells were located in T_EMRA_ cells of patients with SPMS, whereas there was no prominently increase of GzmB expression monitored in RRMS (Fig. 4A–C). We also compared the GzmB expression of CD8 + T cells and their subsets in treated and untreated MS patients, but no significant differences were found (Additional file 1: Fig. S4). To further look into the GZMB distribution in T8EM and T8EMRA subclusters, annotation gene sets analysis was employed to identify correlation patterns of multi genes (Fig. 4D, E). Except a significantly elevation of GZMB expression in T8EM2 subclusters, comparable GZMB levels were observed in T8EM1, T8EM1 and T8EM4 subclusters between patients with RRMS and SPMS (Fig. 4D). Nevertheless, T8EMRA1 and T8EMRA2 subclusters from SPMS patients appeared marked raising of GZMB expression in comparing with RRMS patients (Fig. 4E). As the unique subcluster only detected in SPMS patients, T8EMRA-SP also showed high level of GZMB. Collectively, exclusively GZMB increasing in T8EMRA-SP, T8EMRA1 and T8EMRA2 with clonal expansion may correlated with disease transition of patients with SPMS.

### T-bet manipulates GzmB expression in CD8 + T cells

**Fig. 5:**
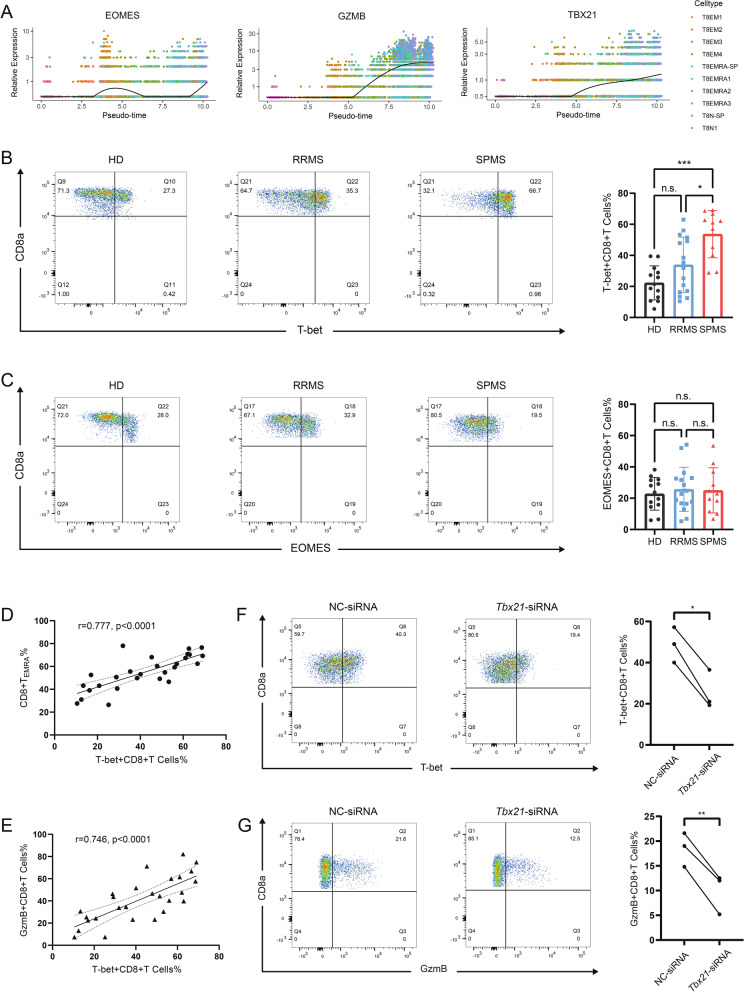
Up-regulation of transcription factor T-bet is associated with high expression of GzmB in circulating CD8 + T cells. **A** Relative expressions of GZMB, TBX21, and EOMES in CD8 + T subclusters were analyzed by pseudo-time analysis. **B**, **C** The proportions of T-bet and EOMES in CD8 + T cells among HD (*n* = 14), RRMS patients (*n* = 16), and SPMS (*n* = 11) were measured by flow cytometry. **D**, **E** The correlation between T-bet expression and the proportion of CD8 + T_EMRA_ cells (**D**) or GzmB + CD8 + T cells (**E**). **F, G** Knock-down the expression of *Tbx21* in CD8 + T cells from SPMS patients using siRNA (*n* = 3). The expression of T-bet (**F**) as well as GzmB (**G**) were significantly eliminated. ***P < 0.001, **P < 0.01, *P < 0.05, n.s. (not significant)

Due to the substantial proportion of CD8 + T_EMRA_ cells expressed GzmB, a key cytokine reminiscent of cytotoxic function, we investigated whether effector CD8 + T cell-fate decision transcription factor *Tbx21* and *Eomes* give rise to *Gzmb* elevating in CD8 + T cells from SMPS patients. Pseudo-time analysis revealed that TBX21, but not EOMES was transcriptionally similar with GZMB (Fig. 5A). Simultaneously, results from involved MS patients showed constitutively up-regulation of T-bet in SPMS patients by comparing with HD and RRMS patients, whereas comparable EOMES levels were observed among HD, RRMS and SPMS patients (Fig. 5B, C). Following spearman correlation analysis revealed concurrent trends in T-bet expression with elevating of CD8 + T_EMRA_ cell proportion and increased GzmB expression (Fig. 5D, E. Thus, by lacking of Eomes expression, peripheral CD8 + T cells from SPMS patients with high levels of T-bet were phenotypically identical to CD8 + T_EMRA_ cells defined by CD45RA + and CCR7- (Figs. 2G and 5D). To interrogate whether GzmB expression was attributed to T-bet, siRNA was then performed to knock-down the expression of *Tbx21* in CD8 + T cells from SPMS patients (Fig. 5F). In comply with decreasing of T-bet levels after RNA interference, GzmB expression was detected to be significantly eliminated in cultured primary human CD8 + T cells as well (Fig. 5G). Overall, this finding identified that T-bet acts as a key transcriptional factor for eliciting GzmB expression in expanded CD8 + T_EMRA_ cells of patients with SPMS.

### Intrinsic GzmB expression in CD8 + T cells is required for disabilities of patients with MS

**Fig. 6:**
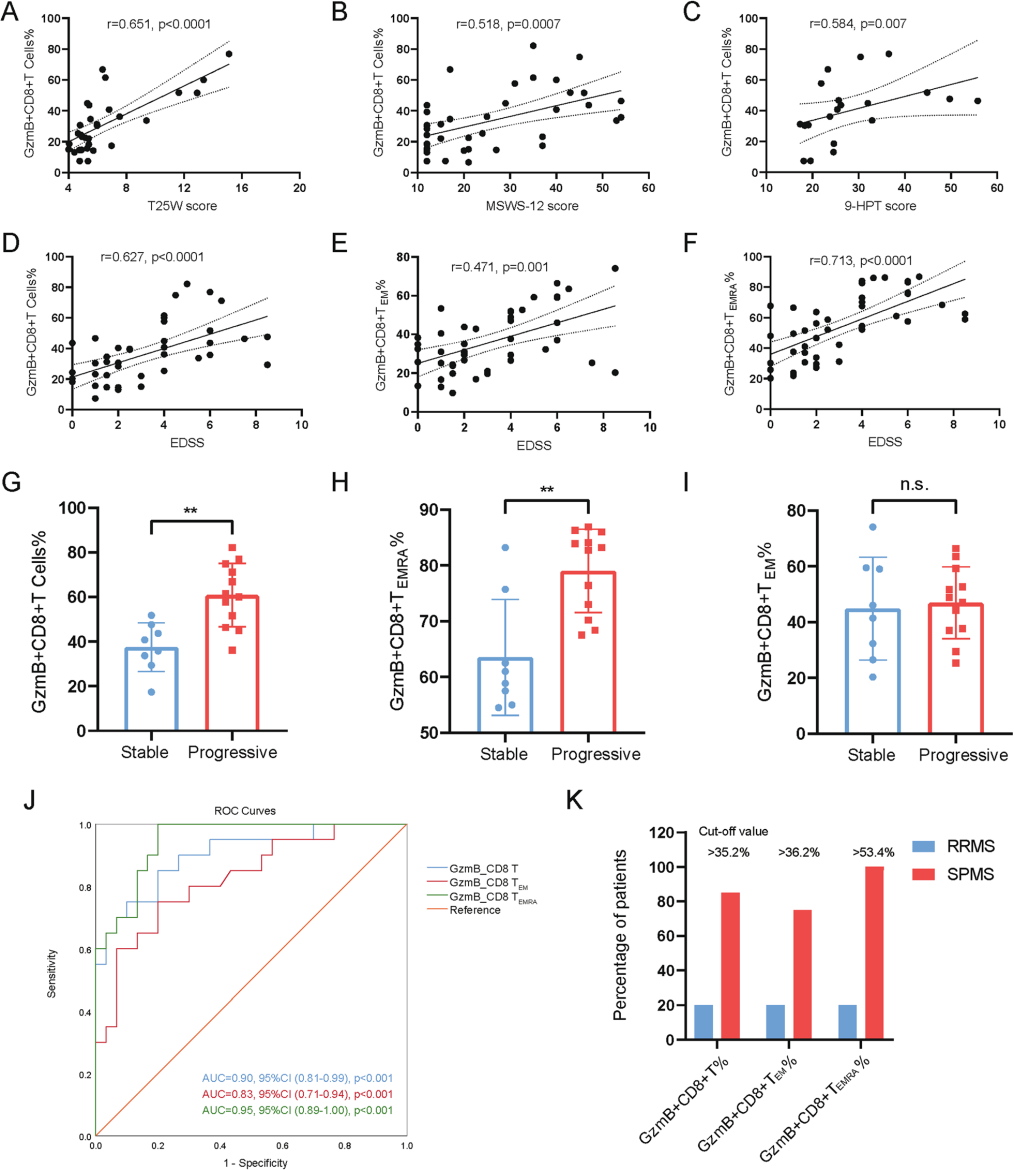
Proportion of GzmB + CD8 + T cells and GzmB + CD8 + T_EMRA_ cells could be used to distinguish SPMS from RRMS. **A**–**C** Positive correlation of GzmB + CD8 + T cells with T25W score (**A**), MSWS-12 score (**B**) and 9-HT score (**C**) were found. **D**–**F** Strong correlation between EDSS scores and the percentage of GzmB + CD8 + T (**D**), GzmB + CD8 + T_EM_ (**E**), as well as GzmB + CD8 + T_EMRA_. **F** were found in MS, while there was a moderate correlation between EDSS scores and the percentage of GzmB + CD8 + T_EM_ cells_._ **G**–**I** SPMS patients were divided into stable group or progressive group according to the EDSS scores in the past year. The progressive group showed significantly higher expression of GzmB in CD8 + T cells (**G**) and CD8 + T_EMRA_ cells (**I**) but not in CD8 + T_EM_ cells (**H**). **J** The proportion of GzmB + CD8 + T cells and GzmB + CD8 + T_EMRA_ cells showed good discriminative ability to distinguish SPMS from RRMS. **K** The cut-off values of GzmB expression of SPMS prediction was obtained from ROC curve, which result in a low false positive rate and true positive rate to diagnose SPMS. **P < 0.01, n.s. (not significant)

Considering the increasing trend in proportion of peripheral GzmB + CD8 + T cells, we resorted to investigate the relationship between GzmB levels in peripheral CD8 + T cells and limb disabilities of patients. Increasing of peripheral GzmB + CD8 + T cell proportions were closely correlated with severities of limb disabilities (T25W, *r* = 0.651, *P* < 0.001; MSWS-12, *r* = 0.497, *P* = 0.002; 9-HPT, *r* = 0.553, *P* = 0.009) (Fig. 6A–C). As the most well-recognized assessment in evaluating MS patients, EDSS scores were employed to explore the association between disabilities and peripheral GzmB + CD8 + T cells, GzmB + CD8 + T_EM_ cells, or GzmB + CD8 + T_EMRA_ cells, respectively. Accordingly, all CD8 + T cell subsets that expressed GzmB were positively related to EDSS scores from patients with MS, especially GzmB + CD8 + T_EMRA_ cells, which exhibited best-fitting correlation with severities of MS patients (Fig. 6D–F). Hence, as an easily accessible parameter, elevating of GzmB + CD8 + T_EMRA_ cell proportion in peripheral emerge as an independent risk factor for evaluating the severity of MS patients with progressive stages.

### Elevation of GzmB particularly in CD8 + TEMRA cells contributes to SPMS progression

Next, we divided SPMS patients into “stable” and “progressive” states based on EDSS score changes over the past year. Since disease were prominently progressed, frequencies of both GzmB + CD8 + T cells and GzmB + CD8 + T_EMRA_ cells significantly increased, but not GzmB + CD8 + T_EM_ subset (Fig. 6G–I). This result prompted us to further dissect the potently predictive features of GzmB + CD8 + T cells and/or GzmB + CD8 + T_EMRA_ for SPMS progression. Other than the association of GzmB + CD8 + T_EM_ cells with age, disease duration and SPMS subtypes, dynamic changes of GzmB + CD8 + T cell and GzmB + CD8 + T_EMRA_ cell proportions were only positively correlated with SPMS, respectively (Additional file 3: Tables S2–S4). While percentages of GzmB + CD8 + T_EM_ seemed to be associated with age, disease duration, and SPMS subtypes (Additional file 3: Table S3). Following diagnostic capability analysis of GzmB + subsets in determining SPMS were performed, the estimation of the area under the curve (AUC) improved to 95.3% (*P* < 0.001) in circulating GzmB + CD8 + T_EMRA_ cells group, whereas GzmB + CD8 + T and GzmB + CD8 + T_EM_ cells presented relatively lower AUC of 94.3% (*P* < 0.001) and 76.6% (*P* = 0.003), respectively (Fig. 6J). The cut-off value to distinguish RRMS and SPMS was 35.2% for GzmB + CD8 + T cell percentage, 36.2% for GzmB + CD8 + T_EM_ cell percentage, and 53.4% for GzmB + CD8 + T_EMRA_ cell percentage, respectively (Fig. 6K). Taken together, these results link the unique subcluster of GzmB + CD8 + T_EMRA_ cells, which was mainly derived from clonal expansion in SPMS patients, may serve as a potential diagnostic marker for monitoring SPMS transition at early period.

## Discussion

In recent decades, understanding of immune mechanism in RRMS development has led to the applications of multiple DMT, which opens a window of opportunities for MS treatment [4]. Nevertheless, investigation of SPMS is comparatively disappointing, and few therapeutic approach is proved effective in progressive MS up to date [2]. On the other hand, diagnosis of SPMS is also challenging for both patient and physician due to most of the evidences are obtained retrospectively and delayed [7]. Therefore, instead of this evaluation regarding indolent nature symptom progression, it is an urgent issue in searching reliable real-time diagnostic markers for SPMS transition at clinical practices that ultimately impacting patient management and treatment [6]. By comparing mutually exclusive atlases of peripheral immune cells in patients with remitting or secondary progressive stages, we determined the unique CD8 + T_EMRA_ cells from clonal expansion in disease progression (Figs. 1 and 3). In addition, following studies revealed that accumulation of peripheral GzmB + CD8 + T_EMRA_ cells in SPMS patients compared to those with RRMS (Figs. 2 and 4). Meanwhile, in consistent with previously studies that T-bet/Eomes centered transcriptional network drives effector versus exhausted CD8 + T cell-fate decision [30], T-bet was further confirmed to be responsible for GzmB expression in CD8 + T cells (Fig. 5). Furthermore, the unique alternation from GzmB + CD8 + T_EMRA_ cells to progressive phenotype of MS and its significantly correlation to EDSS underline a possibility in dynamically diagnosing SPMS from active stages of RRMS (Fig. 6).

In considering most of the pathological evidences in MS progression are from lesion at autopsies that mainly reflect the end stage of disease development [16, 31], clinical evaluations and structural imaging are the widely used assessments for finding discrepancy between RRMS and SPMS, which also bring over 10% of misclassification rate in routinely practice [32]. Therefore, the differential diagnosis for determining transition from RRMS to SPMS in pathological aspects would facilitate contemporary diagnostic process and therapeutic decisions for dynamic changes of MS [33]. For a long time, gray matter demyelination, axonal loss and neuronal death are believed to underlie the degeneration happening at late stage of MS progression [11, 33]. Accordingly, biomarkers that likely reflect neurotoxicity, gliosis and CNS destruction are thought to distinguish RRMS and SPMS [10]. However, slightly degeneration with mild to strong inflammation were reported in most cases of SPMS patients at initial stages [31, 34]. These characteristics of early SPMS bring to a more complicated situation in differential diagnosis without real-time pathological evidences [10]. Here, we concluded the prediction abilities of GzmB + CD8 + T cells, GzmB + CD8 + T_EM_ cells and GzmB + CD8 + T_EMRA_ cells in current data, and peripheral GzmB + CD8 + T_EMRA_ cells from clonal expansion were demonstrated to exhibit the superiority of both sensitivity and specificity for distinguishing SPMS from RRMS (Fig. 6J). Increased functional T_EMRA_ CD8 + T cells in circulating system of SPMS patients are more than ancillary in helping local inflammation at SPMS, but could also be used for determining SPMS transition before degenerative stages. Likewise, as our previously findings in Gillian-Barre Syndrome, which specifically touches peripheral nervous system and may have resemble mechanism of MS onset, peripheral CD8 + T_EM_ and T_EMRA_ cell subsets are observed arising ahead of spontaneous autoimmune neuropathy onset, as well as neurological damage [20]. And latest view in MS believes that the presence of neurological symptoms in patients is accompanied with CD8 + T cell expansion in circulating system [19, 35]. Besides, inhibition of peripheral effector CD8 + T cells are proved to efficiently prevent multiple autoimmune diseases, including Susac syndrome, systemic lupus erythematosus, and inflammatory bowel disease [36–38]. Accordingly, these distinct phenotypes of CD8 + T cell in SPMS patients not only prompted us to further think about its diagnostic probabilities to improve classification rate, but would also be a potentially therapeutic target for preventing disease progression. However, in lacking of longitudinal observations, whether different CD8 + T cell subsets could be used to monitor therapeutic responses in our involved patients after DMT treatments remains an enigma.

Indeed, MS is considered as a chronic inflammation occurring in CNS via autoantigen-triggered specific immune responses, and CD8 + T cells are demonstrated to have close relationship with pathological changes of MS progression [16, 39]. Other than viral infection and antigenic mimicry, peripheral CD8 + T cells are more easily predisposed to differentiate into T_EMRA_ via sequestered autoantigens leakage from CNS during MS progression [40–42]. In addition, this imbalance of peripheral CD8 + T cell differentiation may contribute decisive effects in MS progression due to increased permeability of BBB and T cell infiltrating [15, 43]. Similarly, our trajectories analysis confirmed that SPMS patients possessed large amount of terminal differentiated activating and/or effector CD8 + T cells (T8EMRA-SP subclusters), which directly differentiated from T_EM_ cells (Figs. 2C, D and 3C–E). Previously data in discovering CD8 + T cell differentiation mentioned that TCF-1 drives Eomes and Blimp1 down-regulation to promote memory CD8 + T cell subsets formation and activating [44]. As the down-stream signaling of TCF-1, increased T-bet in T_EM_ CD8 + T cells indicates memorial clonal expansion, terminal differentiation fate, as well as IFNγ, GzmB and Perforin expression [45]. Unfortunately, except T-bet was confirmed to up-regulate GzmB expression in CD8 + T_EMRA_ cells, limited information is obtained from our current scRNAseq data regarding other key genes in this axis (Figs. 1 and 5).

Meanwhile, due to the alternative clonal expansion of CD8 + T_EMRA_ cells regarding TCR repertoire between RRMS and SPMS, we revealed a distinct trajectory of peripheral CD8 + T cell that triggered by TRBV9 and TRAV1-2 (Fig. 3C–E, Additional file 3: Fig. S2). According to previously studies, both epitopes are delivered from CMV, which has already been demonstrated to induce irregular provoking of peripheral CD8 + T_EM_ cells with antigen-independent manner and form memory inflation [46, 47]. In addition, we found that clonal amplification of CD8 + TEMRA in SPMS patients was associated with CMV and EBV, suggesting a potential role of viral infection in MS progression (Additional file 2: Table S2). In considering the close relationship between viral infection and autoimmune neuropathy, it is important to distinguish the functions of expanded that recognized epitopes from virus in peripheral and nerve system. Because only 4 blood samples from MS patients were currently applied to determine TCR diversities of CD8 + T cells in our studies, which might bring inadequate evidence thus impedes us to match the clonal expansion in peripheral with CD8 + T cell accumulation at lesion site during progression of MS. Further investigations should be put to expand the understanding of CD8 + T_EMRA_ origin, differentiation and specificity in disease development. Furthermore, another biological interpretation of peripheral CD8 + T_EMRA_ responsible for MS progression is the formation of tertiary lymphoid follicles, which mediated irreversible damages of neurons and oligodendrocytes [48]. In lacking of paired analysis between circulating system and lesion site, we could not draw the conclusion that infiltrating CD8 + T cells share similar infiltrative paths and differentiated way in our expanded CD8 + T_EMRA_ cells from peripheral system. Tilly G et al. reported that teriflunomide treatment affected CD8 + T memory cells in RRMS patients, but no significant difference of CD8 + T subsets was found between patients treated with or without teriflunomide in our study [49]. Therefore, further longitudinal study is needed to verify the clonal expansion and functional changes of CD8 + T subpopulations in MS patients at different timepoints, and to evaluate the potential impact of DMT.

## Conclusions

In summary, our study mapped peripheral immune cells of RRMS and SPMS patients and provided an evidence for the involvement of cytotoxic CD8 + T_EMRA_ with clonal expansion in MS progression, which could be used as a diagnostic biomarker for distinguishing SPMS from RRMS. Longitudinal study is needed to further clarify the predictive value of cytotoxic CD8 + T_EMRA_ cells in transition diagnosis and prognosis prediction of SPMS at early stages.

## Supplementary Information

**Additional file 1.** Figures S1 to S4.

**Additional file 2.** Table S1.

**Additional file 3.** Tables S2 to S4.

## Data Availability

The data sets used and/or analyzed during this study are available from the corresponding authors on reasonable request.

## Abbreviations

AUC: Area under the curve
BBB: Blood–brain barrier
CM: Central memory
CNS: Central nervous system
DMT: Disease-modifying therapies
EAE: Experimental autoimmune encephalomyelitis (EAE)
EDSS: Expanded disability status scale
Eff: Effector
EM: Effector memory
EMRA: Terminal differentiated effector
FH: Follicular helper
GzmB: Granzyme B
HD: Healthy donors
MS: Multiple sclerosis
N: Naïve
PBMC: Peripheral blood mononuclear cell
ROC: Receiver operating characteristic
RRMS: Relapsing–remitting multiple sclerosis
scRNAseq: Single-cell RNA sequencing
SPMS: Secondary progressive multiple sclerosis
TCR: T-cell receptor
