# Supplementary material for: Granzyme B + CD8 + T cells with terminal differentiated effector signature determine multiple sclerosis progression
Source: J Neuroinflammation. 2023 Jun 2;20:138. doi: 10.1186/s12974-023-02810-0 (PMC10236809; doi:10.1186/s12974-023-02810-0)
Supplement: Supplementary file 1 — Additional file 1. Figures S1 to S4. [file 12974_2023_2810_MOESM1_ESM.docx]

**
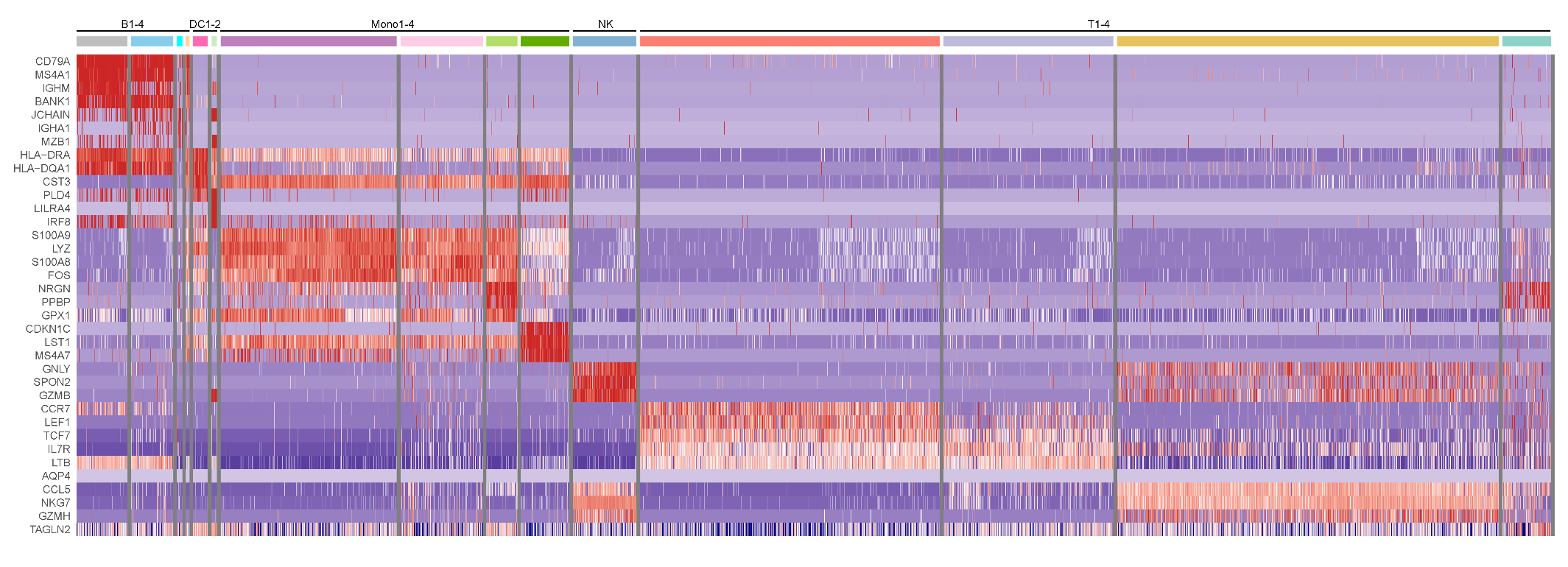
**

**Fig. S1 Heatmap plots of Top 3 differentially expressed genes (DEGs) across all peripheral immune cell clusters.** Non-coding genes are not shown.


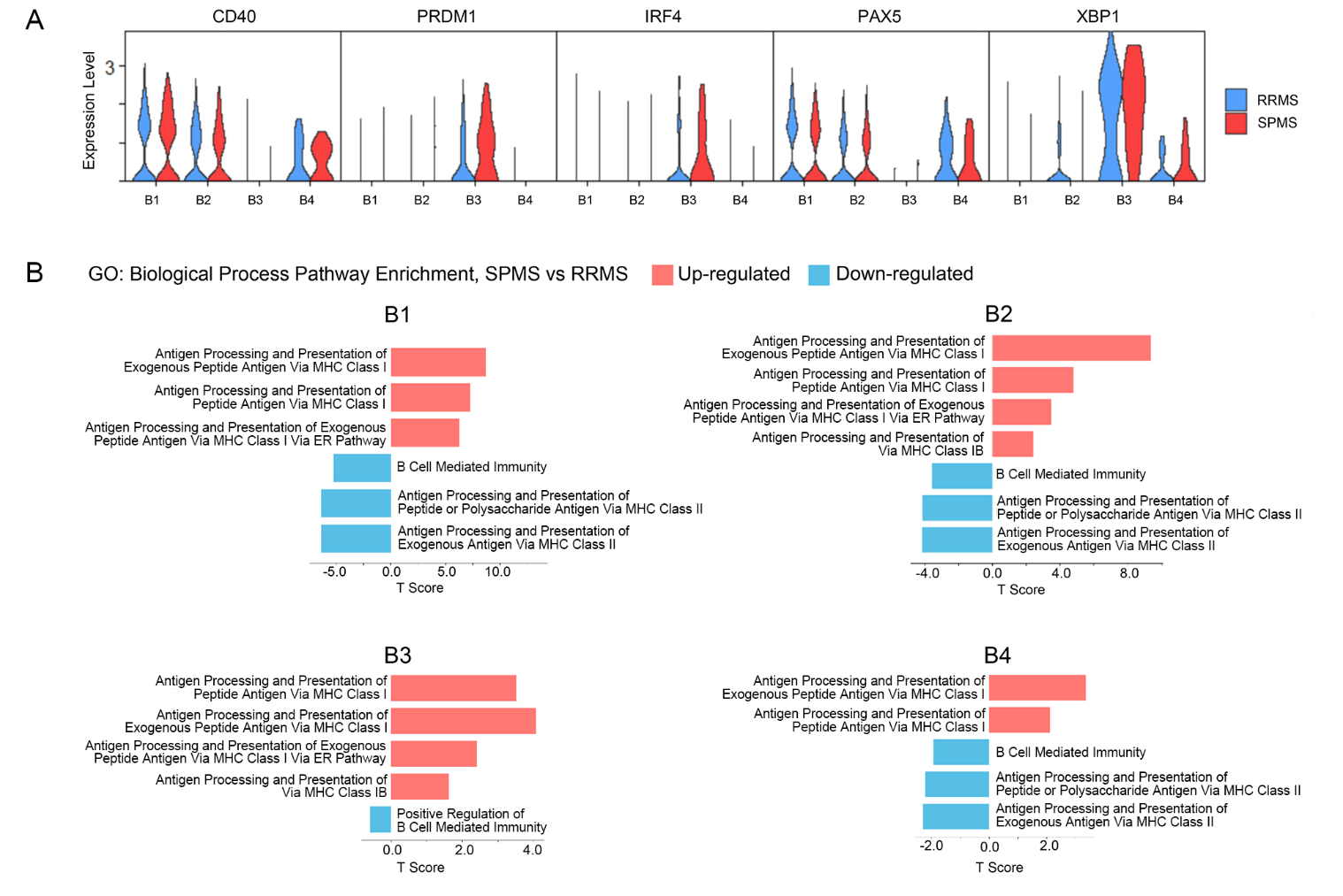


**Fig. S2 Gene expression and function enrichment of B cells in MS.** (A) Expression of functional genes of B cells in RRMS and SPMS. (B) Antigen presenting functions of B cells in SPMS compared with RRMS.

**
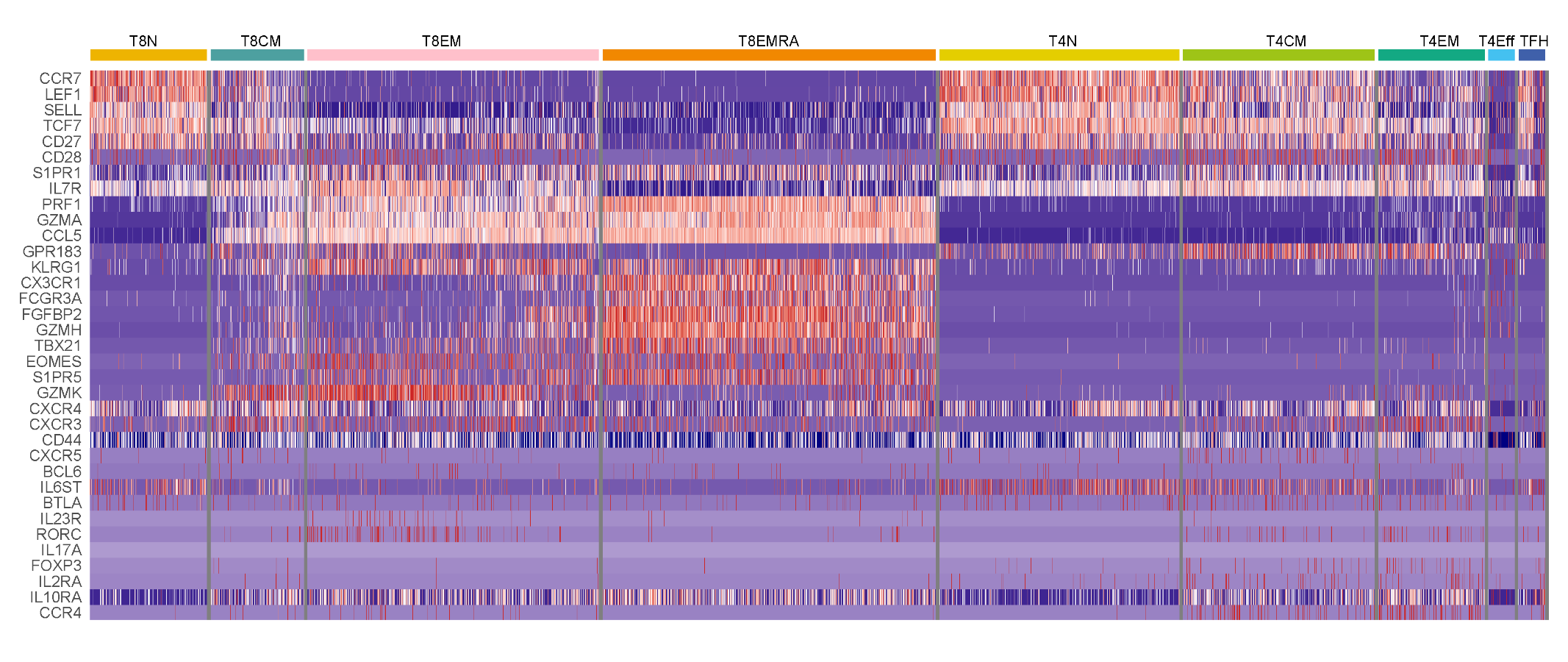
**

**Fig. S3** **Heatmap plots of Top 5 DEGs across T cell subclusters. Non-coding genes are not shown.**


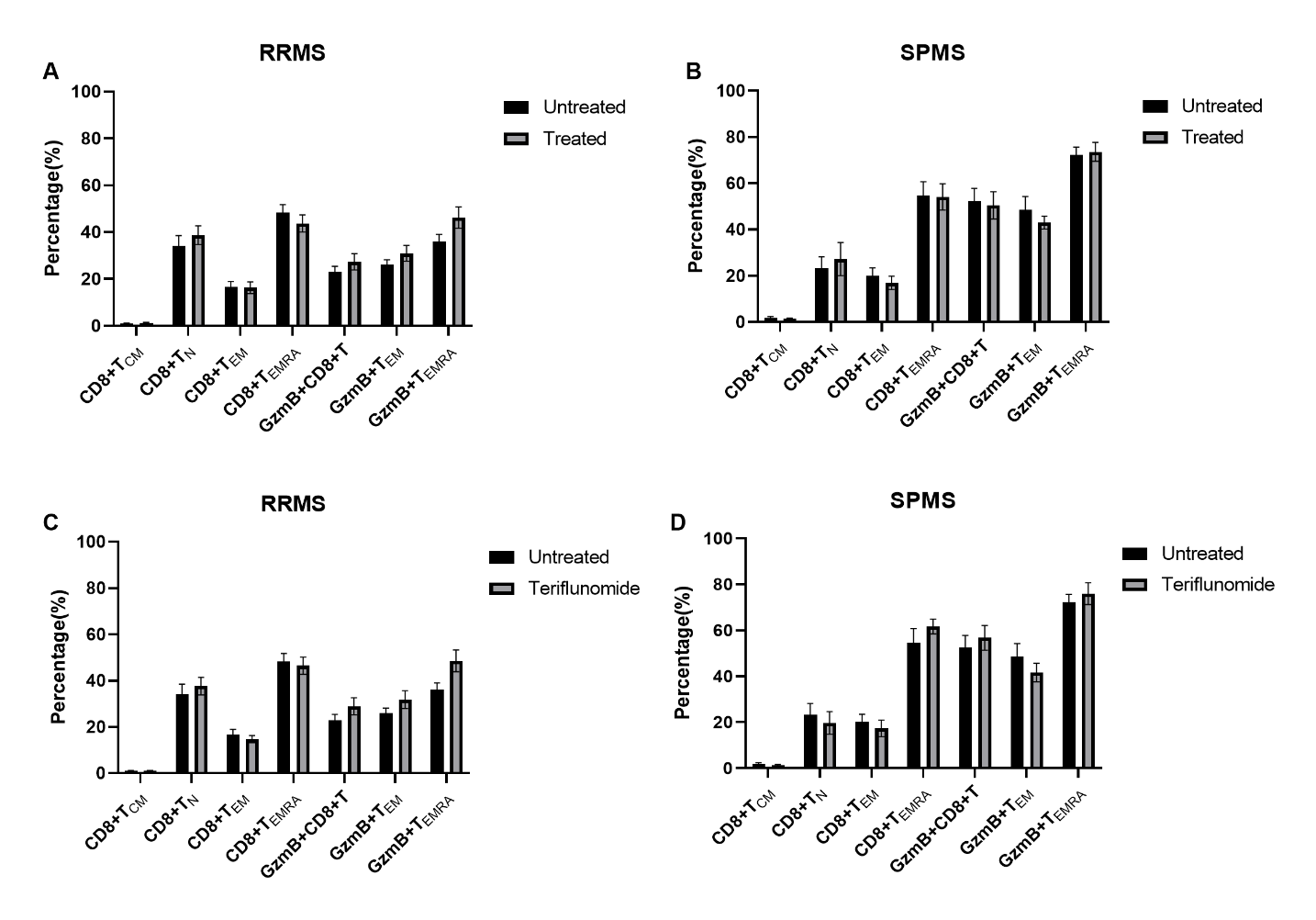


**Fig. S4 Comparison of CD8+T subsets and GzmB expression between treated and untreated MS patients.** RRMS patients, untreated group (n=16) and treated group (n=14, including 12 Teriflunomide and 2 corticosteroids); SPMS patients, untreated group (n=11) and treated group (n=9, including 7 Terifluramide, 1 corticosteroids, and 1 β-IFN). (A) and (B) Percentages of CD8+T subsets (T_CM_, T_N_, T_EM_, T_EMRA_ ) and GzmB expression (GzmB+T, GzmB+T_EM_, GzmB+T_EMRA_) in treated and untreated groups in RRMS and SPMS patients. (C) and (D) Comparison of CD8+T subsets and GzmB expression between untreated patients and Terifluramide treated patients in RRMS and SPMS.
