# Supplementary material for: Granzyme B + CD8 + T cells with terminal differentiated effector signature determine multiple sclerosis progression
Source: J Neuroinflammation. 2023 Jun 2;20:138. doi: 10.1186/s12974-023-02810-0 (PMC10236809; doi:10.1186/s12974-023-02810-0)
Supplement: Supplementary file 3 — Additional file 3. Tables S2 to S4. [file 12974_2023_2810_MOESM3_ESM.docx]

**Table S2. The associations between GzmB expression in CD8+T cells and clinical characteristics in patients with MS.**

|  | GzmB+CD8+T%  Median (IQR) | Univariate analysis | | | | Multivariate analysis | | | |
| --- | --- | --- | --- | --- | --- | --- | --- | --- | --- |
|  |  | β | 95%CI | | *P* value | β | 95%CI | | *P* value |
|  |  |  | Low | Up |  |  | Low | Up |  |
| Sex |  |  |  |  |  |  |  |  |  |
| Female(34) | 30.55 (18.00-41.50) | - | - | - | - | - | - | - | - |
| Male(16) | 44.65 (24.23-50.75) | 9.170 | -1.817 | 20.157 | 0.102 | 5.018 | -3.455 | 13.491 | 0.246 |
| Age, years | - | 0.955 | 0.312 | 1.598 | **0.004** | 0.444 | -0.109 | 0.997 | 0.115 |
| Disease duration, years | - | 0.779 | -0.207 | 1.766 | 0.122 | -0.680 | -1.546 | 0.186 | 0.124 |
| EDSS scores | - | 4.124 | 2.172 | 6.081 | **<0.001** | -0.651 | -3.567 | 2.264 | 0.662 |
| Disease subtype |  |  |  |  |  |  |  |  |  |
| RRMS | 22.85 (14.93-32.13) | - | - | - | - | - | - | - | - |
| SPMS | 49.60 (39.65-63.93) | 26.528 | 18.702 | 34.353 | **<0.001** | 29.320 | 14.334 | 44.305 | **<0.001** |
| Status |  |  |  |  |  |  |  |  |  |
| Non-acute stage | 35.80 (20.30-47.60) | - | - | - | - | - | - | - | - |
| Acute status | 28.40 (15.00-34.60) | -10.442 | -25.323 | 4.440 | 0.169 | 1.765 | -10.118 | 13.649 | 0.771 |
| Treatments^a^ |  | -2.478 | -13.194 | 8.238 | 0.650 | 0.505 | -7.732 | 8.742 | 0.904 |
| Treated | 36.20 (18.60-46.70) | - | - | - | - | - | - | - | - |
| Untreated | 31.30 (20.30-46.40) | -2.478 | -13.194 | 8.238 | 0.650 | 0.505 | -7.732 | 8.742 | 0.904 |

IQR = Interquartile range; CI = confidence interval; EDSS = Expanded Disability Status Scale; RRMS = Relapse-remission multiple sclerosis; SPMS = Secondary progressive multiple sclerosis; ^a^Treatments include β-IFN, Teriflunomide, and/or Corticosteroids. Generalized linear regression models were used for Univariate and Multivariate analysis.

**Table S3. The associations between GzmB expression in CD8+ T_EM_ cells and clinical characteristics in patients with MS.**

|  | GzmB+CD8+T_EM_%  Median (IQR) | Univariate analysis | | | | Multivariate analysis | | | |
| --- | --- | --- | --- | --- | --- | --- | --- | --- | --- |
|  |  | β | 95%CI | | *P* value | β | 95%CI | | *P* value |
|  |  |  | Low | Up |  |  | Low | Up |  |
| Sex |  |  |  |  |  |  |  |  |  |
| Female(34) | 29.40 (22.38-40.73) | - | - | - | - | - | - | - | - |
| Male(16) | 43.30 (29.05-53.23) | 10.592 | 2.103 | 19.081 | **0.014** | 4.299 | -2.262 | 10.861 | 0.199 |
| Age, years | - | -0.232 | -0.782 | 0.318 | 0.408 | -0.644 | -1.072 | -0.216 | **0.003** |
| Disease duration, years | - | 0.186 | -0.617 | 0.989 | 0.650 | -0.913 | -1.584 | -0.242 | **0.008** |
| EDSS scores | - | 3.460 | 1.933 | 4.988 | **<0.001** | 1.512 | -0.745 | 3.770 | 0.189 |
| Disease subtype |  |  |  |  |  |  |  |  |  |
| RRMS | 26.90 (20.70-34.95) | - | - | - | - | - | - | - | - |
| SPMS | 46.55 (33.48-59.15) | 17.753 | 10.757 | 24.748 | **0.001** | 19.382 | 7.777 | 30.986 | **0.001** |
| Status |  |  |  |  |  |  |  |  |  |
| Non-acute stage | 32.50 (25.20-48.80) | - | - | - | - | - | - | - | - |
| Acute status | 27.50 (23.80-35.10) | -7.338 | -19.241 | 4.565 | 0.227 | -1.755 | -10.958 | 7.447 | 0.709 |
| Treatment |  |  |  |  |  |  |  |  |  |
| Treated | 37.00 (26.50-47.10) | - | - | - | - | - | - | - | - |
| Untreated | 29.40 (22.40-46.00) | -1.067 | -9.615 | 7.481 | 0.807 | 0.080 | -6.298 | 6.458 | 0.980 |

IQR = Interquartile range; CI = confidence interval; EDSS = Expanded Disability Status Scale; RRMS = Relapse-remission multiple sclerosis; SPMS = Secondary progressive multiple sclerosis; ^a^Treatments include β-IFN, Teriflunomide, and/or Corticosteroids. Generalized linear regression models were used for Univariate and Multivariate analysis.

**Table S4. The associations between GzmB expression in CD8+T_EMRA_ cells and clinical characteristics in patients with MS**

|  | GzmB+CD8+T_EMRA_%  Median (IQR) | Univariate analysis | | | | Multivariate analysis | | | |
| --- | --- | --- | --- | --- | --- | --- | --- | --- | --- |
|  |  | β | 95%CI | | *P* value | β | 95%CI | | *P* value |
|  |  |  | Low | Up |  |  | Low | Up |  |
| Sex |  |  |  |  |  |  |  |  |  |
| Female | 50.50 (30.58-64.65) | - | - | - | - | - | - | - | - |
| Male | 67.45 (42.43-83.20) | 14.579 | 2.905 | 26.252 | **0.014** | 6.996 | -1.001 | 14.994 | 0.086 |
| Age, years | - | 0.831 | 0.104 | 1.557 | **0.025** | 0.132 | -0.390 | 0.654 | 0.620 |
| Disease duration, years | - | 0.954 | -0.121 | 2.028 | 0.082 | -0.587 | -1.404 | 0.231 | 0.160 |
| EDSS scores | - | 5.000 | 2.943 | 7.057 | **<0.001** | -0.439 | -3.191 | 2.312 | 0.754 |
| Disease subtype |  |  |  |  |  |  |  |  |  |
| RRMS | 39.25 (27.35-52.13) | - | - | - | - | - | - | - | - |
| SPMS | 74.35 (61.38-83.73) | 32.035 | 24.320 | 39.750 | **<0.001** | 34.948 | 20.804 | 49.092 | **<0.001** |
| Status |  |  |  |  |  |  |  |  |  |
| Non-acute stage | 57.50 (37.00-73.00) | - | - | - | - | - | - | - | - |
| Acute status | 41.10 (29.60-51.50) | -14.829 | -30.919 | 1.262 | 0.071 | 1.685 | -9.531 | 12.901 | 0.768 |
| Treatment |  |  |  |  |  |  |  |  |  |
| Treated | 55.00 (42.30-73.00) | - | - | - | - | - | - | - | - |
| Untreated | 49.50 (30.20-68.40) | -8.768 | -20.277 | 2.740 | 0.135 | -6.219 | -13.993 | 1.555 | 0.117 |

IQR = Interquartile range; CI = confidence interval; EDSS = Expanded Disability Status Scale; RRMS = Relapse-remission multiple sclerosis; SPMS = Secondary progressive multiple sclerosis; ^a^Treatments include β-IFN, Teriflunomide, and/or Corticosteroids. Generalized linear regresion models were used for Univariate and Multivariate analysis.
